# To the Editors of the Medical and Physical Journal

**Published:** 1800-05

**Authors:** John Ring

**Affiliations:** New Street, Hanover Square


### *To the Editors of the Medical and Phyſical Journal.*

GENTLEMEN,

IF you ſhould think the following Luſus Naturæ as ſingular as I do, you will probably give it a place in your uſeful Journal. I am, GENTLEMEN,

Your obedient humble ſervant,

### *To the Editors of the Medical and Phyſical Journal.*

GENTLEMEN,

IN the Britiſh Magazine for March appeared a caſe of Catalepſy, drawn up by the gentleman who was the ſubject of the diſeaſe. He conceals his name, and ſigns Medicus, not chuſing to encounter

——— " the world's dread laugh,

Which ſcarce the firm philoſopher can ſcorn."

The exiſtence of this diſeaſe was doubted by Cullen; but even Cullen was not infallible. I have known one inſtance of it, it, which occurred in the family of Mr. Butler, late of Conduit Street, now of Bruton Street, about four years ago.

In this cafe there can be no reafon to fufpect impofition, either from the characters of the parties who defcribed the diforder to me, or the circumftances of the patient.

Mr. and Mrs. Butler fent for me in great hafte, but before I could arrive the paroxyfm was over. They informed me, that in whatever attitude the patient was, when attacked, fhe remained perfectly motionlefs about ten minutes. She had feveral of thefe attacks before I faw her, at intervals of a few weeks; and I expreffed a wifh to fee her, if poffible, whenever another paroxyfm fhould occur; but Mr. and Mrs. B. were fearful that an accident might happen, if fhe fhould have a fit when carrying a candle, and difmiffed her on that account; to the mutual regret of both parties. They gave me an excellent character of her as a fervant; and it is impoffible to conceive that fhe would counterfeit a difeafe, which muft occafion the lofs of an indulgent mafter and miftrefs, and a good place.

I mentioned this cafe at the Medical Society; and Mr. Hurlock faid it was confirmed to him by the teftimony of the mother; who faid, fhe was then in an hofpital on account of it, not being able to earn her living. I am,

Gentlemen,

Your moft obedient fervant,

JOHN RING.

*New Street, Hanover Square,*
*April* 18, 1800.

